# Molecular docking of resveratrol with known protein structures as well as high-throughput meta-data analyses uncork the “French Paradox” and its potential “Druggable” targets in humans

**DOI:** 10.1186/1471-2164-15-S2-P27

**Published:** 2014-04-02

**Authors:** Peter Natesan Pushparaj, Zeenat Mirza, Sajjad Karim, Kalamegam Gauthaman, Kothandaraman Narasimhan, Adel Abuzenadah, Mamdooh Gari, Adeel Chaudhary, Mohammed Alqahtani

**Affiliations:** 1 Center of Excellence in Genomic Medicine Research, Faculty of Applied Medical Sciences, King Abdulaziz University, Jeddah 21589, Kingdom of Saudi Arabia; 2 King Fahd Medical Research Center, Faculty of Applied Medical Sciences, King Abdulaziz University, Jeddah 21589, Kingdom of Saudi Arabia; 3 Department of Medical Laboratory Technology, Faculty of Applied Medical Sciences, King Abdulaziz University, Kingdom of Saudi Arabia

## Background

Resveratrol (RSV) is a polyphenolic phytoalexin (3, 5, 4'-trihydroxystilbene) produced by plants as a protective defense mechanism against pathogens or environmental stress [1]. RSV is found in various plant species such as peanuts, mulberries, Japanese knotweed and skin of grapes. As such RSV is a component of red wine. Interestingly, the prevalence of coronary heart disease (CHD) is comparatively reduced in people from Southern France despite high dietary intake of saturated fats [1-3]. In order to uncork this “French-Paradox”, as well as to identify other potential health benefits of RSV in relation to cancer, infection and inflammation, we studied molecular docking of RSV with known protein structures implicated in health and disease and further dissected the potential molecular networks regulated by RSV.

## Materials and methods

TarFishDock Server (http://www.dddc.ac.cn/tarfisdock) was used to dock RSV with 698 known drug target proteins in the Potential Drug Target Database (PDTB). This automated webserver enables docking of small molecules with a broad range of Protein Structures available in the Protein Drug Target Database (PDTD) (http://www.dddc.ac.cn/pdtd/) [4]. Furthermore, to define biological networks and dissect the functional relationship among the genes regulated by RSV, we performed pathway analyses using the Ingenuity Pathways Analysis (IPA) (Ingenuity Systems, Redwood City, CA, USA). The significance of the association of RSV with gene networks was calculated by ratio and/or Fisher’s exact.

## Results

RSV docking with known protein structures has uncovered its potential to bind with proteins (Table 1) such as Matrix Metalloproteinase-8 (MMP8), Nicotinate-nucleotide Adenylyltransferase, Myeloperoxidase (MPO), Oxidosqualene cyclase, Nitric Oxide Synthase (NOS) etc.,. These proteins are commonly implicated in cancer, cardiovascular diseases, multiple sclerosis, infection, inflammation, sepsis and diabetic retinopathy. Additionally, the IPA analyses have revealed that a total of 350 genes were either associated or regulated by RSV in health and disease.

## Conclusions

Our study has identified potential molecular networks regulated by RSV in cancer, cardiovascular diseases, infection, inflammation and neurodegenerative disorders. RSV is reported to cause vasorelaxation and improve myocardial function by increasing endothelial nitric oxide synthase, and thereby substantially uncork the “French Paradox”. Besides, the present study offers a method to deduce the suitability of small molecules such as RSV for clinical trials for specific disease pathology. Conversely, precise docking with “Druggable” target proteins identified in our current study would certainly be essential to obtain comprehensive information to design RSV analogues for the treatment of an array of diseases afflicting humans in the near future.
